# Prevalence and factors associated with gonorrhea infection with respect to anatomic distributions among men who have sex with men

**DOI:** 10.1371/journal.pone.0211682

**Published:** 2019-04-03

**Authors:** Jiratha Budkaew, Bandit Chumworathayi, Chamsai Pientong, Tipaya Ekalaksananan

**Affiliations:** 1 Family Physician, Department of Social Medicine, Khon Kaen Center Hospital, Khon Kaen Province, Thailand; 2 Gynecologic Oncologist, Department of Obstetrics and Gynecology, Faculty of Medicine, Srinagarind Hospital, Khon Kaen University, Thailand; 3 Generalist, Department of Microbiology, Faculty of Medicine, Srinagarind Hospital, Khon Kaen University, Thailand; 4 Family Physician, Department of Microbiology, Faculty of Medicine, Srinagarind Hospital, Khon Kaen University, Thailand; Agencia de Salut Publica de Barcelona, SPAIN

## Abstract

**Introduction:**

Gonorrhea (GC) infection caused by *Neisseria gonorrhoeae* has been steadily increasing in Thailand over the last decade. Men who have sex with men (MSM) are at high risk for gonorrhea infection.

**Materials and methods:**

In this study, we determined the prevalence of and risk factors associated with gonococcal infections by three anatomical sites among MSM. We have conducted a cross-sectional analysis of a sexually transmitted disease (STD), gonorrhea among MSM attending two STD clinics in Khon Kaen, Thailand. We included 358 MSM over 18 years of age. Data were collected using self-administered questionnaire. In each participant, an oropharyngeal, anorectal, and endourethral swab were tested with culture and nucleic acid amplification test (NAAT). However, 267 urine samples were tested by both methods. Factors associated with gonorrhea infections were assessed using univariate and multivariate logistic regression.

**Results:**

One hundred and ninety-five out of 358 (54.47%) MSM tested were found to be positive for gonorrhea using a porA gene targeted NAAT by Real-time PCR with TaqMan probes, but there was no positive result by culture. The gonorrheal prevalence for male genital site, anal, and oropharyngeal, were 34.73% (95%CI 33.07, 45.08), 29.01% (95%CI 24.61, 34.33), and 27.93% (95%CI 23.35, 32.89), respectively, while 5.9% (21/355) were positive for gonococcal infection in all anatomic sites (oropharynx + anus + urethra) of one participant. Previous history of diagnosed STDs was a significant factor associated urethral gonorrhea (odds ratio = 3.52, 95%CI 1.87–6.66, P Value< 0.001). In addition, having more than one partner was increased urethral gonorrhea (adjusted odds ratio = 2.26, 95%CI 1.10–4.68, P Value = 0.026). 100% of condom use was found decreasing urethral infection (adjusted odds ratio = 0.39, 95%CI 0.15–0.99, P Value = 0.046).

**Conclusions:**

The most common anatomic site of gonorrhea infection was male genital site, and the independent risk factors were having history of diagnosed STDs and having more than one partner in the past 3 months, but 100% condom use was a protective factor of this infection.

## Introduction

Gonorrhea (GC) caused by *Neisseria Gonorrhoeae* is a high prevalent sexually transmitted disease (STD) in less-developed countries and lower [1] and it is still substantial and increasing rates of disease in many developed countries [2]. GC is treatable with administration of appropriate antibiotics albeit problem of antibiotic resistance is rising [3]. Symptoms of gonorrhea are yellowish discharge from penis, burning sensation, dysuria, anal discharge and anal itching, erythematous exudate of pharynx, and sore throat [2]. Asymptomatic gonorrhea is significantly common in men who have sex with men (MSM) which remains undiagnosed and untreated and may lead to a reservoir which can result in widespread transmission among multiple partners [4]. In extra-genital sites, oropharyngeal and rectal infections are mostly asymptomatic and may be important in gonorrheal transmission among MSM [5–6].

The prevalence of this infection varies by anatomic sites (urethral, rectal, and oropharyngeal) [6] and the detection methods (gram’s stain, standard culture, and molecular test (Nucleic Acid Amplification Tests; NAATs) [7]. The sensitivity of standard culture (the traditional gold standard) is greatly decreased at rectal and oropharyngeal sites [8–9]. This leads culture to an unacceptable first line diagnostic or confirmatory test for *N*. *gonorrhoeae* at extra-genital sites, and cases of gonorrhea may be missed. There are a range of NAAT tests available for the detection of *N*. *gonorrhoeae*, not all have a high sensitivity and specificity, particularly for extra-genital sites [10].

There are many factors found associated with high prevalence rate of gonorrhea infection such as unsafe sex [2], HIV sero-positive [11], multiple partners [11], previous diagnosed STDs [12]. However, there are various risk factors associated with this infection are not clearly known among MSM including drug/ alcohol use [13], younger age [14], payment or receive for sex [15], symptoms of gonorrhea [13], and history of their partner STDs [16]. Knowledge of risk factors plays an important role in designing effective control measures and they are presumptive indicators for therapy [17]. For instance, knowledge about how often MSM using condom during sex may help physicians for accurate risk assessment and counseling of persons at risk on ways to avoid STDs through changes in sexual behaviors and use of recommended prevention services.

Thailand is a major area for STDs such as gonorrhea because of the growth of sex industry leading more and more antibiotic resistance [18]. The prevalence of gonorrhea among asymptomatic MSM was 6.1%, most frequent affected the anus [19]. Sex education has been the largest contribution to fighting STDs in Thailand. In fact, a Thailand national survey revealed 50 percent of those surveyed did not wear condoms when engaging in sexual intercourse [20]. Screening for asymptomatic MSM has become standard of care in many developed countries but has not occurred in many developing countries, including Thailand [18]. Thus, the purpose of this study was to determine the prevalence of and risk factors associated with GC infections by anatomical sites among asymptomatic MSM.

## Materials and methods

### Study population

In August 2015 until May 2016, a cross-sectional study of MSM considered to be at high risk for gonorrhea infection was initiated in a research clinic. MSM aged ≥18 years were eligible if they met any of the following criteria: reported having anal intercourse either insertive anal intercourse (IAI) or receptive anal intercourse (RAI) [21] in their lifetime, and did not take any antibiotics during the previous two weeks. Participants were categorized as symptomatic if they presented with one or more of the following: dysuria, urogenital bleeding, pelvic or genital pain, urethral discharge, genital lesions, genital itching or rash, or urethritis, anal pain, itching, anal discharge, sore throat and redness of pharynx, discomfort when swallowing, whitish/ yellowish discharge in the oropharyngeal area. Participants not exhibiting any of these symptoms were classified as asymptomatic. MSM were excluded if they were taking treatment for a recent STD or had taken antibiotics such treatment in the past two weeks prior to study screening.

### Recruitment

MSM were recruited from two walk-in clinics by using the combination of snowball sampling method (for some who did not willing to identify themselves as MSM) and direct recruitment (for some who willing to identify themselves as MSM). The first clinic was a sexually transmitted diseases mobile clinic (STDs mobile clinic), and an antiretroviral clinic (ARV clinic) both a part of Khon Kaen Hospital, located in Khon Kaen in the northeast of Thailand. In addition, identification and recruitment data of participants were collected by the first author who approached individuals via personal networks of participants and at social venues. The social venues were selected as recruitment sites by Local M-REACH team. They selected the big outdoor events where MSM had joined such as Thai traditional dance, Loi-Kratong festival, etc. by using time-location sampling. Recruitment activities encompassed a region of the Khon Kaen municipality area. Meetings were held with local M-REACH teams (a Non-government organization supported by the collaboration between the Thai and United States governments to prevent sexually infection diseases among MSM) to enlist support for the ongoing research and to prevent misunderstanding among the study population. Verbal informed consent was obtained from all participants. The study protocol was approval by the Khon Kaen Hospital Ethics Committee for Human Research and the Khon Kaen University Ethics Committee for Human Research.

### Study activities

Participants were informed verbal and written explanation about the purpose, procedures, potential risks and benefits of the study. In our study, both IRBs allowed waiver of documentation of consent (if the participant is given all the relevant information and has been asked for consent verbally, but a written consent document is not used, then documentation has been waived) [22] for all subjects. Enrolled participants attended the STDs clinic one time. At the clinic, participants completed a self-administered questionnaire that asked about recent sex behavior in the past 3 months, including number of partners, alcohol or drug use before or during sex activity. Participants were requested their HIV status, and some of them were tested for HIV depending on their will. Participants were asked whether they had a dysuria, pus or yellowish discharge at penis, pruritus at anus or sore throat at the visit or having trauma (abrasion or lacerated wound) at sexual contacting organ during sex. Symptoms of urethral, anal and oropharyngeal pain, discharge, itch or irritation were assessed. A clinical examination to identify STDs and HIV-related clinical conditions was performed by a qualified clinician with experience in providing health care to MSM to reassure that they had symptom or not. Participants also provided self-collected urine and a clinician collected oropharyngeal, rectal and urethral swabs.

After one or two weeks, participants were informed of their specimen results by telephone, if they were diagnosed with infections, they were treated with appropriate antibiotics according to the Thailand National guidelines recommended by CDC [23]. Gonorrhea was treated with a dual regimen which is a combination of ceftriazone 250 mg intramuscular (IM) as a single dose for gonorrhea and azitromycin 1 gram orally as single dose or doxycyclin 100 mg orally twice a day for 7 days for treatment for chlamydial, co-infection [24]. Sexual contacts were traced for treatment and where necessary patients were linked back to the clinic for relevant care. Participants received sexual risk-reduction counseling, condoms and latex-compatible sexual lubricants and a range of information and educational materials.

The data are neither ethically or legally restricted, and are not third-party data. The dataset file (1-358GC-MSMs-DataSet) is available from the Dryad database at URL http://datadryad.org/review?doi=doi:10.5061/dryad.9r06k.

### Laboratory analysis

#### Screening for gonorrhea

**Oropharyngeal swab** We performed oropharyngeal swab by pushing the tongue downwards with a spatula or hold it with fingers and gauze, wiping the posterior wall and the tonsils with the swab so that as much of cells as possible are collected from the mucosal surface, avoiding contact with the mucous of the cheek or the tongue in order to collect as much sample from the throat as possible, and cutting the swab with scissors into a sample tube at about 1–2 cm from the nylon head of the swab.

**Rectal swab** The rectal swab was performed by inserting sterile swab approximately 1–1.5 inches in the anal canal, moving swab from side to side in the anal canal to sample crypts, and allowing swab to remain 10–30 seconds for absorption of organisms onto the swab.

**Urethral swab** Discharge from the meatus is preferred for the detection of *N gonorrhea*. If there is no meatal exudate in postpubertal male, an endourethral swab can be used for the detection of gonococci. To increase the chance of detecting the organisms, swab samples should be collected from participants who have not voided for at least 2 hrs.

The swabs from urethral, rectal, and oropharyngeal are suitable for smear preparation, culturing on appropriate media or for transport to other laboratories.

**Urine** Leak-proof containers should be provided to participants for the collection of urine specimens. All samples were tested via NAAT, but urine specimen should not be used for gonorrheal culture due to its low sensitivity.

In each participant, an oro-pharyngeal, rectal, and endourethral swab were taken separately by gently passing each cotton tipped swab 1–4 cm inside the urethral meatus and rotated it by 360°. One swab for each site was collected. After being smeared on a glass slide for microscopy and plated for culture, it was not discarded, but was placed in 2SP (sucrose phosphate) transport medium for NAAT. A 10–30 ml sample of first voided urine was collected after the swabs. The Real‐time PCR with TaqMan probes was performed to detect *N*. *gonorrhoeae* DNA.

The prevalence of urethral gonorrhea was detected by both urine collection and urethral swab. At first, when we developed the proposal and conducted the research, we collected sample from urethral swab, but after reviewing in more literatures, we found that urine sample is one of an important sample to detect gonorrhea. Therefore, fewer urine samples were tested [25].

**Conventional culture** The Laboratory of Microbiology Department of Srinagarind Hospital performed susceptibility testing of all isolates of *N*. *gonorrhoeae* by means of disc testing following the **Clinical and Laboratory Standards Institute (CLSI)** guidelines. The specimens were inoculated onto MTM agar plates immediately (clinical diagnostics LTD, Thailand) and incubated for 24–48 hours at 37°C in 5% CO_2_ or under anaerobic conditions. Plates were examined after 18 hours of incubation, and if the result was negative, they were repeatedly examined after 24 hours of incubation [26]. Morphologically suggestive colonies *of N*. *gonorrhoeae* were further processed for confirmation by means of Gram staining, oxidase and glucose utilization tests.

**Real Time Polymerase Chain Reaction (Real-time PCR with TaqMan probes)** The gonococcal porA pseudogene is a popular target for in-house *N*. *gonorrhoeae* PCR methods. It has previously been shown to be highly conserved and specific to *N*. *gonorrhoeae* [27]. For real-time PCR analysis, 2 μl of extracted DNA samples were performed. The TaqMan real-time PCR reaction mixture contained variable amounts of total DNA, the forward primer, reverse primer, TaqMan^Ò^ probe, and TaqMan Universal PCR Master Mix. Forward primer was 5’-CAGCATTCAATTTGTTCCGAGTC-3’. Reverse primer was 5’-GAACTGGTTTCATCTGATTACTTTCCA-3’. The specific TaqMan^Ò^ probe for *Neisseria gonorrhoeae* detection was 5’-CGCCTATACGCCTGCTACTTTCACGC-3’. The thermal cycle conditions for TaqMan^Ò^ assay were as follows: 10 min at 95°C, 10 sec at 95°C, 30 sec at 60°C and 10 sec at 72°C 40 cycles. The amplification plot used to define the threshold cycle (Ct) for a sample. Gel electrophoresis was confirmed the real-time PCR product with 89 bp. The sensitivity of NAATs for the detection of *N*. *gonorrhea* is superior to culture. The sensitivity was 100%, with specificities of 99.3% and 98.8%, respectively [26].

#### Data analysis

Data were analyzed using Stata 12.0 (Stata Corporation, College Station, TX, USA). Descriptive statistics (proportions, means with SD, medians with IQR) were used to summarize the data. We conducted bivariate and multivariate logistic regression analyses reporting the estimated odds ratios, adjusted odds, and their 95% confidence intervals and χ2 tests to explore associations between main effect variables and GC status. Logistic regression is used to predict a categorical (usually dichotomous) variable from a set of predictor variables. Predictors of STD were assessed using a logistic regression model. The associated odds ratios (OR) and the corresponding 95% confidence limits were reported. The variables included in the multivariable logistic regression model were first assessed for their association with the outcome of interest using a bivariate logistic regression model. If the reported P Value associated with that variable in the univariate model was below 0.25, then it qualified to be included in the multivariable model. McNemar test was used to explore associations between GC status and other factors.

## Results

We recruited a total of 358 people. No one was screened and found that he is ineligible. The median age was 28 years old (minimum–maximum: 18–60 years) among those who were included in this study and whose data were analyzed (Table 1). Three participants refused to provide anal and urethral swabs. For urethral gonorrhea, we firstly performed urethral swab, after reviewing in more detail, the evidence indicated that urine is one of an appropriated sample for gonorrhea infection. Then, 267 participants collected their urine. More than half (52.79%) had both insertive and receptive anal sexual behavior and were employed (58.10%). Thirty-one percent (31.84%) of participants were diagnosed HIV-positive, while 22.63% had a history of previous STDs. The majority of participants were asymptomatic. They attended two STD clinics for regular check up every 3 months. Three participants (less than 1%) indicated they had a symptom of gonorrhea. Based on clinical symptoms, the reported sites of infection were urethra and anus. One hundred and ninety eight (55.3%) indicated that they did not know of any history of previous STDs among their partners.

**Table 1:** Participants characteristics, N = 358.

		n	%	Median (IQR)
**Age (years)**				28 (18, 60)
	18–24	127	35.47	
	> = 25	231	64.53	
**Occupation**				
	Employed	208	58.10	
	Non-Employed	150	41.90	
**Basic sex behaviors**				
	Insertive sex	54	15.08	
	Receptive sex	64	17.88	
	Both	189	52.79	
Unknown 51/14.25 **Payment for sex**				
	No	235	65.64	
	Yes	106	29.61	
	Unknown	17	4.75	
**Receipt for sex**				
	No	251	70.11	
	Yes	91	25.42	
	Unknown	16	4.47	
**HIV status**				
	Negative	129	36.03	
	Positive	114	31.84	
	Unknown	115	32.12	
**Number of partners in previous 3 months**				
	None	133	37.15	
	1 partner	127	35.47	
	> 1 partner	98	27.38	
**Having trauma at sexual contacting organ during sex**				
	No	186	51.96	
	Yes	58	16.20	
	Unknown	114	31.84	
**Alcohol before having sex**				
	No	192	53.63	
	Yes	166	46.37	
**Illicit drug use before having sex**				
	No	301	84.08	
	Yes	57	15.92	
**Previous diagnosed STDs**				
	No	277	77.37	
	Yes	81	22.63	
**Condom use (100% use)**				
	No	295	82.40	
	Yes	63	17.60	
**Having gonorrhea symptoms**				
**(discharge in urethra and anus, dysuria, anal pruritus pruritus or sore throat) at least one day in past 3 months**				
	No	355	99.16	
	Yes	3	0.84	
**History of previous STDs of partners**				
	No	145	55.31	
	Yes	15	4.19	
	Unknown	198	55.31	

Sex related characteristics were also assessed. Among them are engagement in sexual intercourse under alcohol and use of illicit drugs, number of partners and use of condom during sex. There were 166 (46.37%) subjects who reported having engaged in sex under the influence of alcohol, while 57 (15.92%) subjects reported that they had engaged in sex under the influence of illicit drugs. Two hundred and twenty-five participants reported to have an active sex life. Of those who had an active sexual life, 127 (35.47%) said that they have had one intimate partner in the last 3 months, and 98 (27.38%) said that they have more than two intimate partners. Nearly one-third (29.7%) of participants reported payment for sex, and about one-fourth (25.42% reported receipt or were paid for sex. Among those participants with reported active sex life indicated that 17.60% always use a condom during sexual intercourse. The results also indicated that 16.20% had trauma which means having abrasion or lacerated wound at the sexual contacting organ during sex.

### GC status by Real-time PCR with TaqMan probes

One hundred and ninety-five MSM (54.78%) out of 358 MSM tested were found to be positive for gonorrhea using a porA gene targeted nucleic acid amplification test (NAAT) by Real-time PCR with TaqMan probes, however, there was no positive result by traditional culture. The gonorrheal prevalence for orophayngeal, anal, and urethral infection were 27.93% (99/358, 95%CI 23.35, 32.89), 29.01% (103/355, 95%CI 24.61, 34.33), and 34.73% (124/357, 95%CI 33.07, 45.08) respectively. The prevalence of urethral gonorrhea was detected from both urethral swab 25.91% (92/355 95%CI 21.96, 31.39) and urine collection was 20.25% (55/269, 95%CI 15.91, 25.95). More than half of MSM (54.78% 95%CI 49.44, 60.03) had detection of gonorrhea in at least one anatomic site. In 5.9% (21/355), GC was found in all anatomic sites.

Because of the fact that GC is often asymptomatic or mild in presentation, we hypothesized that the number of GC cells identified might be associated with symptoms or epidemiological differences. Using the ID50 for GC– 103 organisms–as our cut-off point [28], we divided GC-positive samples into high-level copies (5103) and low-level copies (<103). In this study, low-level positive GC samples were detected which is not given that most of participants were symptom free.

Table 2 shows the basic characteristics by anatomic sites of positive Real-time PCR with TaqMan probes results. MSM > 25 years old had a higher infection rate than the younger age group. In the group of employed MSM infection with gonorrhea was higher than in the unemployed group, and participants who identified both as having receptive and insertive anal sex was the most infected people. MSM who pay and/ or were paid (receipt) for sex was less likely to be infected with gonorrhea than those who did not pay or receipt for sex. MSM who are HIV positive had similar infection rates of gonorrhea compared to those who are HIV-negative or with unknown HIV status, except in urethral infection. Surprisingly, participants who reported having one partner had a higher infection rate than those either have no partner or who have multiple partners. Participants reported using condom (100% use), drinking alcohol before having sex, having illicit drug use before having sex, having trauma at the sexual contact organ during sex, and having history of previous STDs had infection rate of gonorrhea less than those who had not. Two participants (< 1%) who reported having symptoms were infected with gonorrhea at both urethra and the anorectal site.

**Table 2:** Basic characteristics of participants by anatomic distributions of positive results using PCR with TaqMan probes.

		Oropharyngeal		Urethra		Anus		
		n = 100	%	n = 126	%	n = 104	%	P Value
**Age (years**)								0.89
	18–24	41	41.00	45	35.71	32	30.77	
	> = 25	59	59.00	81	64.29	72	69.23	
**Median (IQR):**		24 (18, 45)		29 (18, 60)		26 (18, 54)		
**Occupation**							0.02*	
	Employed	61	61.00	79	62.70	69	66.35	
	Non-Employed	39	39.00	47	37.30	35	33.65	
**Basic sex behaviors**								0.32
	Insertive sex	20	20.00	14	11.11	14	13.46	
	Receptive sex	21	21.00	29	23.02	18	17.31	
	Both	44	44.00	64	50.79	54	51.92	
	Unknown	15	15.00	19	15.08	18	17.31	
**Payment for sex**								0.44
	No	69	69.00	92	73.02	68	65.38	
	Yes	29	29.00	28	22.22	27	25.96	
	Unknown	2	2.00	6	4.76	9	8.65	
**Receive for sex**								0.81
	No	68	68.00	93	73.81	71	68.27	
	Yes	31	31.00	28	22.22	27	25.96	
	Unknown	1	1.00	5	3.97	6	5.77	
**HIV status**								0.64
	Negative	36	36.00	36	28.57	40	38.46	
	Positive	31	31.00	52	41.27	37	35.58	
	Unknown	33	33.00	38	30.16	27	25.96	
**Number of partners in previous 3 months**								0.23
	None	33	33.00	43	34.13	36	34.62	
	1 partner	42	42.00	50	39.68	38	36.54	
	> 1 partner	25	25.00	33	26.19	30	28.85	
**Having trauma at sexual contacting organ during sex**								0.97
	No	52	52.00	64	50.79	59	56.73	
	Yes	17	17.00	17	13.49	19	18.27	
	Unknown	31	31.00	45	35.71	26	25.00	
**Alcohol before having sex**								0.71
	No	56	56.00	75	59.52	51	49.04	
	Yes	44	44.00	51	40.48	53	50.96	
**Illicit drug use before having sex**								0.27
	No	82	82.00	112	88.89	92	88.46	
	Yes	18	18.00	14	11.11	12	11.54	
**Previous diagnosed STDs**								0.04*
	No	78	78.00	88	69.84	75	72.12	
	Yes	22	22.00	38	30.16	29	27.88	
**Condom use (100% use)**								0.98
	No	87	87.00	97	76.98	87	83.65	
	Yes	13	13.00	29	23.02	17	16.35	
**Having gonorrhea symptoms**								
**(discharge in urethra and anus, dysuria, anal pruritus pruritus or sore throat) at least one day in past 3 months**								0.64
	No	100	100.00	124	98.41	102	98.08	
	Yes	0	0.00	2	1.59	2	1.92	
**History of previous STDs of partners**								0.77
	No	43	43.00	51	40.48	38	36.54	
	Yes	6	6.00	5	3.97	5	4.81	
	Unknown	51	51.00	70	55.56	61	58.65	

Table 3 shows the univariate association between the variables and the outcome by 3 anatomic sites of gonorrheal infection. The variable, previous history of diagnosed STDs by the respondent met the threshold for inclusion in the multivariable logistic regression model analysis (P Value = 0.006), however when assessing with this model, the result was not associated with the outcome. The test for association between two variables, condom use and illicit drug use, and the outcome were done using a binary regression model. The results showed that these variables were slightly associated with the outcome of urethral site (OR: 0.56, 95% CI: 0.27, 1.15, P Value = 0.097 and OR: 0.67, 95% CI: 0.47, 1.07, P Value = 0.099 respectively). Number of partners in previous 3 months, having trauma at sexual contacting organ during sex and history of previous STDs of partners were also included in the multivariable regression model analysis though there was no evidence of association between these variables and the outcome. These variables were included because they were considered to be important confounders.

**Table 3:** Odds ratio for each selected factors associated with gonorrhea by anatomic distributions using univariate analysis.

Variables	Gonorrhea infection by anatomic sites								
	Oropharynx			Urethra			Anus		
	OR	95% CI	P Value	OR	95% CI	P Value	OR	95% CI	P Value
**Age (years)**			0.175			0.873			0.232
- > = 25	0.72	0.45, 1.16		0.96	0.59, 1.57		1.35	0.83, 2.20	
**Occupation**			0.273			0.721			0.314
- Employed	0.86	0.49, 1.48		1.10	0.65, 1.85		0.86	0.44, 1.31	
**Basic sex behaviors**			0.231			0.331			0.786
- Insertive sex	1.41	0.62, 3.20		0.72	0.30, 1.74		0.68	0.29, 1.56	
- Receptive sex	1.17	0.53, 2.60		1.29	0.58, 2.85		0.73	0.33, 1.62	
- Both	0.73	0.37, 1.45		0.75	0.38, 1.49		0.73	0.38, 1.41	
**Occupation** - Employed	0.86	0.49, 1.48	0.273	1.10	0.65, 1.85	0.721	0.86	0.44, 1.31	0.314
**Payment for sex**			0.527			0.126			0.563
- Yes	1.17	0.23, 2.11		0.95	0.33, 2.23		1.19	0.25, 2.13	
**Receipt for sex**			0.561			0.124			0.452
- Yes	1.27	0.22, 2.32		0.94	0.30, 2.18		1.38	0.31, 2.45	
**HIV status**			0.901			0.463			0.804
- Positive	1.04	0.59, 1.82		0.81	0.46, 1.43		0.93	0.54, 1.61	
**Number of partners in previous 3 months**			0.278			0.284			0.836
- 1 partner	1.50	0.87, 2.57		1.48	0.86		1.13	0.66, 1.93	
- > 1 partner	1.04	0.57, 1.89		1.00	0.54, 1.86		1.18	0.66, 2.10	
**Having trauma at sexual contacting organ during sex**			0.842			0.879			0.839
**- Yes**	1.07	0.56, 2.05		0.95	0.47, 1.92		1.07	0.57, 2.01	
**Alcohol before having sex**			0.576			0.169			0.276
- Yes	0.88	0.55, 1.39		0.72	0.44, 1.15		1.29	0.82, 2.04	
**Illicit drug use before having sex**			0.508			0.099*			0.162
- Yes	1.23	0.67, 2.28		0.56	0.27, 1.15		0.613	0.31, 1.22	
**Previous diagnosed STDs**			0.860			0.006**			0.122
- Yes	0.95	0.55, 1.66		2.13	1.25, 3.62		1.52	0.89, 2.57	
**Condom use (100% use)**			0.891			0.097*			0.658
- Yes	1.03	0.65, 1.65		0.67	0.42, 1.07		0.90	0.57, 1.43	
**Having gonorrhea symptoms** **(discharge in urethra and anus, dysuria, anal pruritus pruritus or sore throat) at least one day in past 3 months**			0.314			0.273			0.721
- Yes	0.86	0.44, 1.31		0.85	0.49, 1.48		1.10	0.65, 1.85	
**History of previous STDs of partners**			0.418			0.738			0.577
- Yes	1.58	0.53, 4.72		0.80	0.21, 3.01		1.38	0.44, 4.30	

The results in Table 4 show two variables that were significantly associated with urethral gonorrhea are previous history of diagnosed STDs and having more than one partner in the past 3 months (AOR: 3.52 (95% CI: 1.87–6.66, P Value<0.001, and AOR 2.26, 95%CI: 1.10–4.68, P Value = 0.026). On the other hand, previous illicit drug use before having sex, 100% condom use, having trauma at sexual contacting organ during sex, and history of previous STDs of partners were found not statistically significant.

**Table 4:** Adjusted odds ratio for each selected factors associated with gonorrhea by anatomic distributions using multivariate analysis.

Variables	Gonorrhea infection by anatomic sites								
	Oropharynx			Urethra			Anus		
	OR	95%CI	P Value	OR	95%CI	P Value	OR	95%CI	PValue
**Previous diagnosed STDs**			0.775			<0.001*			0.216
- Yes	1.10	0.58–2.06		3.52	1.87–6.66		1.47	0.80–2.71	
**Number of partners in previous 3 months**									
- 1 partner	0.89	0.45–1.77	0.741	1.58	0.73–3.43	0.250	1.17	0.60–2.28	0.646
- 2+ partner	1.44	0.77–2.70	0.255	2.26	1.10–4.68	0.026*	1.18	0.63–2.22	0.611
**Illicit drug use before having sex**			0.599			0.128			0.149
- Yes	1.19	0.62–2.29		0.52	0.23–1.21		0.58	0.28–1.21	
**Condom use (100% use)**			0.428			0.508			0.480
- Yes	0.80	0.46–1.39		0.81	0.44–1.50		0.82	0.47–1.43	
**Having trauma at sexual contacting organ during sex**			0.863			0.470			0.887
- Yes	0.94	0.46–1.92		0.74	0.32–1.69		1.05	0.52–2.14	
**History of previous STDs of partners**			0.266			0.203			0.760
- Yes	1.94	0.60–6.22		0.39	0.09–1.66		1.21	0.36–4.02	

The results in Table 5 indicate one variable that was significantly associated with the outcome is condom use (100% use) which points in the direction of reduced risk. The odds ratio and the corresponding 95% confidence interval (95% CI) was AOR: 0.39 (95% CI: 0.15–0.99, P Value = 0.046). Other variables including previous illicit drug use before having sex, history of previous STDs of partners, history of diagnosed STDs, number of partners in previous 3 months, and having trauma at sexual contacting organ during sex were not statistically significant in this model but all point in the direction of increased risk. The reason why we do this analysis approach is to determine if any infections in one person at any sites associated with which specific risk factor.

**Table 5:** Adjusted odds ratio for each selected factors associated with gonorrhea by total anatomic sites using multivariate analysis.

Variables	OR	95%CI	P Value
**Previous diagnosed STDs**			
- Yes	1.78	0.55, 5.79	0.338
**Number of partners in previous 3 months**			
- 1 partner	1.81	0.68, 4.81	0.416
- 2+ partner	2.02	0.52, 7.83	
**Illicit drug use before having sex**			
- Yes	1.43	0.53, 3.67	0.062
**Condom use (100% use)**			
- Yes	0.39	0.15, 0.99	0.046**
**Having trauma at sexual contacting organ during sex**			
- Yes	0.53	0.17, 1.69	0.282
**History of previous STDs of partners**			
- Yes	0.19	0.03, 1.24	0.083

## Discussion

This is the first study to report the prevalence of gonorrhea infection at an STD and ARV clinic in Khon Kaen Hospital by molecular testing. Diagnosis of any STDs among persons practicing risky sexual behaviors, and particularly persons infected with HIV has significant public health consequences. If these people and their partners continue their risky behaviors (*e*.*g*., inconsistent condom use with all partners), they may increase spreading of STDs and HIV infection [29]. Therefore, routine screening for STDs should be done for early detection and adequate treatment whether they have symptoms or not.

At first, we aimed to use two methods, traditional culture and Real-time PCR with TaqMan probes, for detection of gonorrhea. Surprisingly, there was no positive result on culture, thus we could not analyze drug sensitivity and the pattern of multidrug resistance. This finding was concordant with multicenter study among asymptomatic male which indicated low positive result by culture (prevalence = 1.6%) [30]. Therefore this traditional method could not be a gold standard for screening or even detecting of gonorrhea in asymptomatic MSM.

Testing men and women who are sexually active and less 18–24 years of age each year, 10% of infected males [31] and 80% of infected females are asymptomatic [31]. However, in 2011, one study reported that over 80% of males were asymptomatic [32]. Two of these studies had conflicting data. Gonorrheal infection in urogenital site is more frequently symptomatic than asymptomatic. On the other hand, gonorrheal infection in oropharyngeal and anorectum may be asymptomatic more often than symptomatic [33]. One study showed a high prevalence (7 percent) of asymptomatic rectal gonorrhea in MSM [16]. Asymptomatic gonorrhea in MSM remains undiagnosed and untreated and may lead to a reservoir and which can result in wide spread of transmission among multiple partners [4]. The number of GC cells identified might be associated with symptoms or epidemiological differences. Using the ID50 for GC– 103 organisms–as our cut-off point tested by Real-time PCR with TaqMan probes, the researchers divided GC-positive samples into high-level copies (5103) and low-level copies (<103) [12]. This means that if infected MSM has low level of GC cell in their anatomic sites, they could not develop any symptoms of gonorrhea infection, but they could transmit their bacterial to partners by sexual contact. In 1987, Potterat et al estimated that approximately 35% of gonorrhea transmitted from men is from those who are asymptomatic [34]. Moreover, the diagnostic methods which have low sensitivity could not identify this infection. Therefore, some techniques such as molecular test which can amplify the number of GC cell or has specific to GC gene might successfully detect gonorrhea infection [35]. Study in San Diego, 15.8% of MSM tested for gonorrhea had a positive test in at least 1 anatomic site, with 38% having a negative urethral test while having a positive test from oropharyngeal or rectal sites by NAATs [36].

In anatomic distribution, gonorrheal infection among MSM distributed by anatomical sites including oropharynx, urethra, and anus is slightly different from infection pattern among men who have sex only with women “which indicated less infected in anal sites [23]. Gonorrheal infection at these sites may be symptomatic or asymptomatic. Asymptomatic infection is more likely to be inadequately diagnosed and treated [37–38]. Most of participants in our study with gonococcal infection reported no symptoms in the genito-urinary, anal, and oropharyngeal areas. The prevalence of asymptomatic infections in this study is consistent with other studies conducted in high sexual activity populations in rural Africa. Among high-risk populations in five countries, the results indicated that between 66.7% and 100% of participants reported as asymptomatic [39]. In addition, the most common site of asymptomatic gonorrhea was found in the extra genital area [6]. In this study, the point prevalence of gonorrhea at the STD and ARV clinic in Khon Kaen Hospital was found to be 54.78% (95%CI 49.44, 60.03) in at least one anatomic site. This result is close to the reported prevalence of GC detection by PCR in of 64% in poor access to medical care area, Kaokoland pastoralist, Namebia where have long been presumed to have high prevalence of gonorrhea [40] and this area is developing country similar to Khon Kaen, Thailand. This finding indicates that the burden of disease has not decreased suggesting that relevant intervention strategies are still important.

The most common sites of infection was found in the genito-urinary area (by urine collection and urethral swab specimens) with a prevalence of 34.73% (95%CI 33.07, 45.08), followed by anorectal and oropharyngeal sites with a prevalence of 29.01% (95%CI 24.61, 34.33) and 27.93% (95%CI 23.35, 32.89) respectively, while 5.9% (21/355) were positive for gonococcal infection in all anatomic sites (oropharynx + anus + urethra) of one participant. These results were slightly different from other studies which indicated high prevalence of asymptomatic gonorrhea at anorectal and oropharyngeal sites [41–42]. These data in our study show that the prevalence of each anatomical site were similar, however the most preferable site for gonorrhea is male genital organ. The main reasons for this may be due to nature of this bacterium in harboring at transitional mucosa specifically found in male genital organ [27]. Another reason, in sexual behavior, male penis is an organ be used for insertive sexual activity to both oropharynx and anus, whereas oral and anus cannot be used together in sexual activity, they are both receptive organs.

The question is why do GC and other STDs under detection, in fact that the prevalence and incidence of STD infection is commonly greatest in areas that lack trained staff and advanced equipment for accurate diagnoses and treatment, including Thailand. Guidelines advocating for empirical STI treatment called syndromic management in asymptomatic high-risk populations have been produced due to the globally high incidence of symptom free STD among MSM [43]. In our setting, syndromic management not only has very limited efficacy [44] in MSM with high infection rates of asymptomatic persons, the bigger problem is probably poor access to care [45]. Even when symptoms are present, seeking treatment is not trivial for people living in a covert society. Homophobia and transphobia display a significant burden of access to healthcare in this group [46].

Males with asymptomatic gonorrhea are important reservoirs for transmission and are at increased risk for developing complications [24]. Therefore, a dual regiment treatment of infected MSM including in men with low copies of bacteria who are carriers to prevent spreading of infection has been done [47]. Antibiotics can successfully cure gonorrhea in adolescents and adults. However, multidrug-resistant strains of gonorrhea are increasing globally [18]. According to the CDC report, there are two reasons for the likely increasing this incidence. First, people may stay infected longer, which increases the chances of spreading it to others. Second, and even more worrisome, they noted that drug-resistant gonorrhea might have mutated to infect people even more easily [48].

For factor associated to GC infection, the results in our study shown in Table 4 indicate that a participant’s previous history of diagnosed STDs was found to associate with urethral gonorrheal infection (AOR: 3.52 (95% CI: 1.87–6.66, P Value<0.001). This finding is concordant with previous studies indicating that asymptomatic GC or CT infection was significantly associated with having a lifetime history of at least one STI infection (OR = 3.69, p<0.02) [12, 49]. On the contrary, knowing history of STDs of partners was slightly associated with gonorrheal infection (p = 0.083). This finding was in accordance with a study that indicated that knowing history of STD of their partners in the past years was significantly associated with reduced risk of getting STDs [16]. It might be because people were more likely to be aware and avoid of having sex with partners they think might have or currently have an STD [50]. Having more than one partner in the past 3 months was also found associated with urethral gonorrhea. The 130 subjects who were positive for gonorrhea infection in this study had an active sexual life with one sexual partner and 88 subjects who reported to have two or more sexual partners. Many studies have indicated that having multiple sexual partners [46, 51–52], as a result, increased probably of encountering an infected partner [53]. More than half (120/195) of infected gonorrhea in our study was among MSM who are sero-positive for HIV. This finding is consistent with the observation made in other studies where there is increased risk of STDs among HIV positive MSM [11, 54].

Condom use (100% use) was the important variable shown significantly associated with the outcome of being positive for gonorrhea (OR: 0.39; 95% CI 0.15, 0.99) in this study shown in Table 5. The analytical results indicate that if a person uses condom during anal sex every time, the odds of having gonorrhea infection is reduced by 61%. This effect was statistically significant at 5% level. Other studies have reported similar findings, where 100% condom use during last sexual act (OR: 0.74, 95% CI 0.15, 0.99), decreased prevalence of GC and CT [55]. Conversely, low condom use during last sexual act, increased prevalence of GC and CT (OR: 1.39, 95%CI 0.51–3.83) [56].

Some studies have suggested that low education level and low socio-economic status are associated with STIs. This has been attributed to risk taking behavior among the people with low education and low socio-economic status [47]. This is discordant with the observation made in this study where there is no association between gonorrheal infection and MSM who are not employed. However, study in Thailand indicated the similar finding that employee was the highest gonorrhea infection group (31.12%) in Thai society [57]. Age was also the variable that was not associated with the outcome of being positive for gonorrhea in this study, although the major case of asymptomatic infection was found in MSM > 25 years old. This finding is concordant with a study in England which indicated that more diagnoses of gonorrhea were reported in MSM aged 25–34 years, with a prevalence of 42% [14]. On the other hand, results from several studies found that MSM who were younger had the majority of new cases of GC/CT infection [58–59].

Payment and/ or being paid for sex were also not associated with gonorrheal infection in our study. These findings were in contrast with many studies that have found that MSM having sexual contact with someone who exchanged sex for money or drugs (OR = 1.82; 95% CI 1.12 to 2.97; p = 0.015) was significantly associated with high rate of GC [38–39]. Some studies mentioned that the damage of mucous membranes commonly occur during sex might have the potential to increase the risk of STDs [60–61].

This is contrast with the finding in our study found that having trauma at sexual contact organ during sex was not associated with gonorrheal infection.

## Study limitations

There are a number of limitations to our study, the main limitation being that our samples obtained by a combination method of registration and snowball sampling of MSM who were purposively recruited from two sexual health services frequented by higher risk MSM and the majority of which had history of a HIV seropositive, which may have influenced our results. It is possible therefore that our self-selected sample may be biased and our findings may not be generalizable to the broader community of MSM.

However, our sample included MSM from different geographic regions, specific settings associated with high risk sexual activity such as social clubs, younger men, and men in HIV clinic settings which may generalize for the population of MSM.

Further limitations, prevalence rate reported here is likely an underestimation given we tested gonorrhea infection in men who experienced asymptomatic infection–Many men may have had symptomatic infection and might not have been recruited. Other limitation, we had collected fewer numbers of urine specimens compared to those from urethral swabs which may affect to the actual prevalence of urethral gonorrhea.

It is important to consider in clinical practice as well as public health campaigns that MSM may be unlikely to reduce sexual behaviors putting them at a higher risk for gonorrhea, or to utilize condoms. From our findings in this study, condom use is the protective factor of gonorrhea infection. This message should be given to public again and again to help decrease all STDs. High-intensity behavioral counseling for all sexually active adolescents and for adults at increased risk of STIs should be implemented to multiple sessions in a primary care or STDs clinic setting. Intensive counseling also increases adherence to treatment in adolescent [62].

## Conclusion

The prevalence of gonorrhea among asymptomatic MSM in this study was very high. The most common site of gonorrhea infection was male genital site, and the independent risk factors for male genital gonorrhea were history of diagnosed STDs and having more than one partner in the past 3 months. On the other hand, 100% condom use was a protective factor of gonorrhea infection in a person. There is needed for increased emphasis on gonorrheal infection screening in all three anatomic sites, among asymptomatic MSM. The NAATs method should be implemented in our setting for higher rate detection of gonorrhea. Health education promoting regular condom use should be continued to prevent risk of gonorrhea infection in populations with risky behavior.

## Supporting information

S1 File Nucleic acid detection of Neisseria gonorrhoeae (protocol). (DOCX)
