# Supplementary material for: Prevalence and factors associated with gonorrhea infection with respect to anatomic distributions among men who have sex with men
Source: PLoS One. 2019 Apr 3;14(4):e0211682. doi: 10.1371/journal.pone.0211682 (PMC6447148; doi:10.1371/journal.pone.0211682)
Supplement: S1 File — (DOCX) [file pone.0211682.s001.docx]

**Nucleic acid detection of *Neisseria gonorrhoeae***

DNA extraction

Oropharyngeal, rectal and anal cells were collected using swabs and kept in sterile formal saline tube (10% formalin with 0.85% sodium chloride). Cells were collected by centrifugation at 2000 rpm (4 xg) for 5 min. and washed 2 times with phosphate buffer saline (PBS). They were lysed using 200 ul of lysis buffer with 15 ul of 50 ul/ml of proteinase K, vortexed until getting clear solution and incubated at 60oC for 30 min. After cooling down at room temperature, the solution was added with 300 ul of protein precipitation, vortexed for 20 sec and centrifuged at 13,500 rpm (177 xg) for 5 min at 4^o^C. The aqueous phase was transferred to a new tube and equal volume of isopropanol was added, stored at -70^o^C for 1 hour. After centrifugation at 13,500 rpm (177 xg) for 5 min at 25^o^C, the supernatant was discarded and the DNA pellet was added with 1 ml of 70% ethanol. The supernatant was removed and the pellet was allowed to be air-dried for 15-30 min. The DNA pellet was resuspended in 40 ul of distilled water and stored at -20 ^o^C until used. DNA quality was determined by detection of GAPDH gene using real-time PCR.

Real time Polymerase Chain Reaction Assays

Extracted material was examined using a slightly modified version of previously published in-house real-time PCR assays which targeted the porA pseudogene (Whiley & Sloots, 2005) and other previous studies (Hjelmevoll et al, and Alexander et al ). TaqMan real time PCR was performed. Probes and primers against the N. gonorrhoeae porA pseudogene were designed according previous report (Whiley & Sloots, 2005) that consisted of probe-papTM-P FAM-CGCCTATACGCCTGCTACTTTCACGC-BHQ1 and the forward and reverse primers were-papTM-F CAGCATTCAATTTGTTCCGAGTC and-papTM-R GAACTGGTTTCATCTGATTACTTTCCA, respectively.

TaqMan real-time PCR assay was performed on LightCycler® 480 Real-Time PCR Instrument (Roche Diagnostic, Germany). The reaction mixture was performed in final volume of 10 µl containing 5 µl SsoAdvanced™ Universal SYBR® Green Supermix (Bio-Rad, Hercules, CA, USA), 0.3 µl of forward primer (300 nM), 0.3 µl of reverse primer (300 nM), 0.5 ul of probe ( 500 nM), 1.9 µl of distilled water and 2 µl of DNA template.

For thermal cycling conditions, DNA detection included initial 3 min. at 95°C for enzyme activation followed by 45 cycles of 95°C, 10 sec. for denaturation and 60°C, 10 sec. for annealing and 72°C 10 sec. for extension. Then, real-time PCR result was confirmed by detection of product amplicon that was electrophoresed in 2% agarose gel.

For positive control in the present study, a suspension of *N. gonorrhoeae* from clinical isolation strain cultured on a modified Thayer-Martin medium and incubated overnight at 37°C in the presence of 5% CO2 was made and counted in phosphate-buffered saline. This was then diluted 10^-1^ through 10^-7^, and extracted for DNA. This DNA was investigated for DNA of *N. gonorrhoeae* by TaqMan real time PCR to evaluate the sensitivity of detection. Similarly, 100 ul of each dilution 10^-1^ through 10^-5^ was transferred to 3 ml of sample transportation buffer and stored overnight at 4°C. The following day, 200 ul of each dilution was used to prepare DNA that used for DNA detection of *N. gonorrhoeae* to estimate the efficacy also of the transportation buffer.

References:

- Whiley, D. M. & Sloots, T. P. (2005). Comparison of three in-house multiplex PCR assays for the detection of Neisseria gonorrhoeae and Chlamydia trachomatis using real-time and conventional detection methodologies. Pathology 37, 364–370.

- Stig Ove Hjelmevoll,* Merethe Elise Olsen,* Johanna U. Ericson Sollid,† Håkon Haaheim* Magnus Unemo,‡ and Vegard Skogen§ , (2006). A Fast Real-Time Polymerase Chain Reaction Method for Sensitive and Specific Detection of the Neisseria gonorrhoeae porA Pseudogene. Journal of Molecular Diagnostics, 8, (5), 574-581, DOI: 10.2353/jmoldx.2006.060024

-Sarah Alexander, Filomeno Coelho da Silva, Rohini Manuel, Rajesh Varma2 and Catherine Ison, (2011). Evaluation of strategies for confirming Neisseria gonorrhoeae nucleic acid amplification tests. Journal of Medical Microbiology, 60, 909–912
